# The research of ARIMA, GM(1,1), and LSTM models for prediction of TB cases in China

**DOI:** 10.1371/journal.pone.0262734

**Published:** 2022-02-23

**Authors:** Daren Zhao, Huiwu Zhang, Qing Cao, Zhiyi Wang, Sizhang He, Minghua Zhou, Ruihua Zhang

**Affiliations:** 1 Department of Medical Administration, Sichuan Provincial Orthopedics Hospital, Chengdu, Sichuan, P.R. China; 2 Department of Medical Administration, Sichuan Academy of Medical Sciences & Sichuan Provincial People’s Hospital, Chengdu, Sichuan, P.R. China; 3 Department of Medical Administration, Sichuan Cancer Hospital & Institute, Chengdu, Sichuan, P.R. China; 4 Department of Information and Statistics, The Affiliated Hospital of Southwest Medical University, Luzhou, Sichuan, P.R. China; 5 Department of Medical Administration, Luzhou People’s Hospital, Luzhou, Sichuan, P.R. China; 6 School of Management, Chengdu University of Traditional Chinese Medicine, Chengdu, Sichuan, P.R. China; Instituto Nacional de Astrofisica Optica y Electronica, MEXICO

## Abstract

**Background and objective:**

Tuberculosis (Tuberculosis, TB) is a public health problem in China, which not only endangers the population’s health but also affects economic and social development. It requires an accurate prediction analysis to help to make policymakers with early warning and provide effective precautionary measures. In this study, ARIMA, GM(1,1), and LSTM models were constructed and compared, respectively. The results showed that the LSTM was the optimal model, which can be achieved satisfactory performance for TB cases predictions in mainland China.

**Methods:**

The data of tuberculosis cases in mainland China were extracted from the National Health Commission of the People’s Republic of China website. According to the TB data characteristics and the sample requirements, we created the ARIMA, GM(1,1), and LSTM models, which can make predictions for the prevalence trend of TB. The mean absolute error (MAE), root mean square error (RMSE), and mean absolute percentage error (MAPE) were applied to evaluate the effects of model fitting predicting accuracy.

**Results:**

There were 3,021,995 tuberculosis cases in mainland China from January 2018 to December 2020. And the overall TB cases in mainland China take on a downtrend trend. We established ARIMA, GM(1,1), and LSTM models, respectively. The optimal ARIMA model is the ARIMA (0,1,0) × (0,1,0)12. The equation for GM(1,1) model was X(k+1) = -10057053.55e^(-0.01k)^ + 10153178.55 the Mean square deviation ratio C value was 0.49, and the Small probability of error P was 0.94. LSTM model consists of an input layer, a hidden layer and an output layer, the parameters of epochs, learning rating are 60, 0.01, respectively. The MAE, RMSE, and MAPE values of LSTM model were smaller than that of GM(1,1) and ARIMA models.

**Conclusions:**

Our findings showed that the LSTM model was the optimal model, which has a higher accuracy performance than that of ARIMA and GM (1,1) models. Its prediction results can act as a predictive tool for TB prevention measures in mainland China.

## Introduction

Tuberculosis, an infectious disease caused by Mycobacterium tuberculosis, is still a major global public health problem [1]. According to the GLOBAL TUBERCULOSIS REPORT 2020 released by the World Health Organization, approximately 10 million people are reported to be infected with TB, among the HIV-negative people, with an estimated 1.2 million people died of TB, and among the HIV-positive people, 208,000 people died of TB [2]. It is classified as a class B infectious disease, and the morbidity and mortality of TB have always been among the top two in the Class A and B infectious diseases in mainland China currently.

If not treated in time, long-term illness could lead to laying a huge economic burden for patients and exerting a stronger influence on social development. There are 30 high TB burden countries account for almost 90% of those who fall sick with TB each year [2]. China has the second largest burden of TB in the world with huge health and economic losses [3].

According to the data from the China Health Statistics Yearbook, the TB mortality rate of China shows an ascendant trend although the TB incidence has been declining for the past few years. Therefore, the prevention and control of TB is still the focus of current research. Meanwhile, a few TB patients have experienced interruptions to their treatment schedules because of the COVID-19 pandemic threatens. Consequently, it is important to realize the early warning in infectious disease surveillance. It is of great significance to create an accurate prediction model for the morbidity of TB and then predict the future epidemic situation, which can provide a basis for scientific guidance on its control and prevention [4].

There are several mathematical methods for infectious disease prediction at present. Through a literature review, the infectious disease prediction model is mainly classified into two categories [5, 6]: the traditional mathematical forecasting model and the machine-learning based forecasting models. The traditional mathematical forecasting models consist of Autoregressive Integrated Moving Average model (ARIMA) [7], Exponential Smoothing model [8], Regression forecast model [9], Grey Markov forecasting model [10], and GM (1,1) model [11] and so on, and the machine-learning based forecasting models, such as BP artificial neural network model [12], Multivariate Adaptive Regression Splines (MARS) [13], Support Vector Machine (SVM) [14], and Long Short-Term Memory (LSTM) [15], etc.

However, different models are suitable for different data characteristics [8]. So there is no doubt that according to the data characteristics and the sample requirements to construct the optimized prediction model, which was a precondition for obtaining accurate prediction performance. Recent studies show that the traditional mathematical forecasting models have performed well in infectious disease prediction. Zheng et al. [4] revealed that ARIMA models are an important tool for infectious disease prediction, and prediction results can help set public health planning by the government in Xinjiang, China. Ilie et al. [16] demonstrated that ARIMA time-series models have been successfully applied in the overall prevalence of COVID-19 in Romania. Wang et al. [17] used ARIMA and GM(1,1) models to predict hepatitis B in China, and the ARIMA model achieved better results than GM(1,1) model. Guo et al. [18] used GM(1,1) and novel SMGM(1,1) models to predict dysentery and gonorrhea in China. Despite the traditional mathematical forecasting models have better ability in infectious disease prediction, these models can not extract nonlinear relationships in time series [15]. However, the machine-learning based forecasting models have good performance in handling nonlinear relationships in time series without any limitations [15].

Yet to date, no researchers have applied the ARIMA, GM(1,1), and LSTM models to predict TB cases in mainland China. Under such a background, the present researcher tried to use the ARIMA, GM(1,1), and LSTM models for prediction of TB cases in mainland China. The TB cases data were obtained from the National Health Commission of the People’s Republic of China website, based on the data characteristics and the sample requirements, we created the ARIMA, GM(1,1), and LSTM models, respectively. In order to achieve more accurate prediction results, three models were compared by MAE, RMSE and MAPE, thus, we find the optimized prediction model to predict the epidemic trend of TB cases from January to December 2021 in mainland China.

## Methods

### Data source

All data TB cases were taken from the National Health Commission of the People’s Republic of China (http://www.nhc.gov.cn/), and all TB cases had been laboratory confirmed. In China, TB is classified as a class B infectious disease, and the hospital physicians must report every confirmed TB case information to the local health authority within 24 hours by national Network report of infectious disease [4], the final report to the National Center for Disease Control and Prevention. The data of TB cases only include in mainland China.

We collected monthly TB cases data from January 2018 to December 2020 with 36 samples in our study. The data can also be divided into a training set, a validation set, and a test set. The training set and the validation set were the same, that was made up of the TB cases from January 2018 to December 2020, which was used to build and compare the ARIMA, GM(1,1) and LSTM models respectively, and the test set constituted the TB cases from January to December in 2021, which was used to assess the performance of prediction results in the future trend of TB cases in mainland China.

### Model descriptions

#### ARIMA model

ARIMA model was proposed by Box and Jenkins in the early 1970s, so it is also called Box-Jenkins model and Box-Jenkins method [19]. It is the most commonly used prediction techniques in the evaluation and monitoring epidemiological surveillance [20–26]. The ARIMA model consists of auto regressive (AR) model, moving average (MA) model, seasonal auto regressive integrated moving average (SARIMA) model and etc [17, 27]. If the data of research showed evidence of seasonal tendency, the seasonal auto regressive integrated moving average (SARIMA) model should be used [28].

In general, the ARIMA model can be explained as ARIMA (p,d,q),×(P,D,Q), where *p* represents the order of auto-regression, *d* represents the degree of trend difference, *q* represents the order of moving average, and the *P* represents the seasonal auto regression lag, *D* represents the degree of seasonal difference, *Q* represents the seasonal moving average, s represents the length of the cyclical pattern [29].

There are several steps to construct the ARIMA model, which mainly contains four steps: sequence stationary, model identification, estimation and diagnosis, and model prediction and evaluation.

*The first step is sequence stationary*. If the sequence shows non-stationary time series, the stationary time series should be transformed by differenceing processes [30]. The original sequence diagram could help suggesting whether the sequence is stationary or not. In order to achieve the stationary time series, differences and Log transformation can be processed by statistical software.

*The second step is model identification*. The preliminary judgment and estimation of ARIMA model parameters are found to be dependent on the auto-correlation function (ACF) and partial auto-correlation function (PACF) graphs. And then, the candidate ARIMA model parameters can be determined by both skills and experiences, observing the auto-correlation function (ACF) and partial auto-correlation function (PACF) graphs.

*The third step is estimation and diagnosis*. The candidate ARIMA models evaluated by the diagnostic checking of residuals with Ljung-Box (Q) test [31], which requires the residual error must be random (significant level p>0.05). If the Q -statistics is less than 0.8, the tentative model is inadequate [4]. In other words, the model should be fitted again. Furthermore, the optimal model was determined by the lowest the Bayesian information criterion of Schwarz (BIC) values and its residual was white noise (significant level p>0.05).

*The forth step is model prediction and evaluation*. The optimal model was applied to predict TB cases from January 2018 to December 2020. And the prediction power were evaluated by comparing the predicted values with actual values [17].

#### GM(1,1) model

GM(1,1) model was proposed by Deng J. L, and it is described a novel mathematical prediction system that can be used to process data which some information is known, unknown and/or incomplete [32]. The steps of the GM(1,1) model are [33–37]:

Assuming that the original sequence shown as:

$${\text{X}}^{\left(0\right)}=\left[{\text{x}}^{\left(0\right)}(1),{\text{x}}^{\left(0\right)}(2),{\text{x}}^{\left(0\right)}(3),\ldots {\text{x}}^{\left(0\right)}(\text{n})\right]$$ (1)

Performing an accumulation to generate a new sequence:

$${\text{X}}^{\left(1\right)}=\left[{\text{x}}^{\left(1\right)}(1),{\text{x}}^{\left(1\right)}(2),{\text{x}}^{\left(1\right)}(3),\ldots {\text{x}}^{\left(1\right)}(\text{n})\right]$$ (2)

Solving adjacent neighbor means by the following formula:

$${\text{Z}}^{\left(1\right)}(\text{t})=\frac{1}{2}\left[{\text{x}}^{\left(1\right)}(\text{t})+{\text{x}}^{\left(1\right)}(\text{t}+1)\right],\text{t}=1,2,3\ldots \text{n}$$ (3)

Establishing first-order linear differential equations by the following formula:

$$\frac{{\text{dx}}^{\left(1\right)}(\text{t})}{\text{dt}}+{\text{ax}}^{\left(1\right)}(\text{t})=\text{b}$$ (4)

Assuming that $\hat{\text{a}}={\left(\text{a},\text{b}\right)}^{\text{T}}$ then identification a can be calculated by the following formula:

$$\hat{\text{a}}={\left(\text{a},\text{b}\right)}^{\text{T}}={\left({\text{B}}^{\text{T}}\text{B}\right)}^{-1}{\text{B}}^{\text{T}}\text{Y}$$ (5)

$$\text{Y}={\left[{\text{x}}^{\left(0\right)}(2),{\text{x}}^{\left(0\right)}(3),\ldots {\text{x}}^{\left(0\right)}(\text{n})\right]}^{\text{T}}$$ (6)

$$\text{B}=\left[\begin{array}{ll} -{\text{z}}^{\left(1\right)}(2) & 1\\ -{\text{z}}^{\left(1\right)}(3) & 1\\ \vdots & \vdots \\ -{\text{z}}^{\left(1\right)}(\text{n}) & 1 \end{array}\right]$$ (7)

The GM(1,1) model can be expressed as:

$${\hat{\text{x}}}^{\left(1\right)}(\text{k}+1)=\frac{\text{b}}{\text{a}}+\left[{\text{x}}^{\left(0\right)}(1)-\frac{\text{b}}{\text{a}}\right]{\text{e}}^{-\text{ak}}\;\text{k}=1,2,3,\ldots \text{n},$$

where *a* is the development coefficient, *b* is the gray effect.

Model test evaluated by the posterior error test:

$$\text{C}=\frac{{\text{S}}_{\text{e}}}{{\text{S}}_{\text{x}}}$$ (8)

$$\text{P}=\text{P}\left\{\varepsilon (\text{k})-\bar{\varepsilon }<0.6745\;{\text{S}}_{\text{x}}\right\}$$ (9)

And *S*_*e*_ represents the standard deviation of the residual sequence, *S*_*x*_ represents the standard deviation of the original sequence and P represents the Small probability of error. The accuracy of the model is shown in Table 1, and the model applicable scope shown as Table 2.

**Table 1:** The accuracy of gray GM (1,1) model.

Prediction accuracy grade	Mean square deviation ratio C	Small probability of error P
Level 1(Excellent)	≤0.35	≥0.95
Level 2 (Qualified)	≤0.50	≥0.80
Level 3 (Barely qualified)	≤0.65	≥0.70
Level 4 (Unqualified)	>0.65	<0.70

**Table 2:** The applicable scope of the GM (1,1) model.

Developing Coefficient a	Prediction Length
-a≤0.3	Medium- and long-term prediction
0.3<-a<0.5	Short-term prediction
0.5<-a<1.0	Modified model to predict
1.0<-a	Not suitable for grey prediction model

#### LSTM model

**Fig 1:**
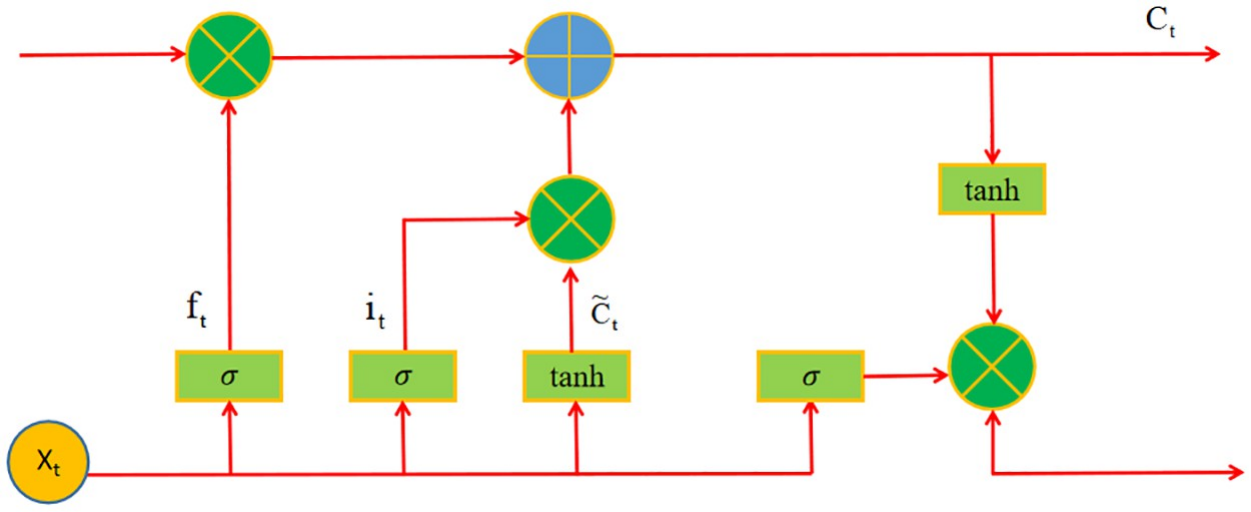
Plot of the LSTM unit structure.

LSTM, called Long Short-Term Memory, has been improved by the Recurrent Neural Network (RNN) [38]. It was first proposed by Hochreiter and Schmidhuber in 1997 and has received the widespread application in many fields [39, 40]. The LSTM unit includes an Input Gate, a Forget Gate, and an Output Gate (Fig 1) [41]. It selectively allows information to pass through Gate structure, so as to update or retain historical information. The LSTM model can be expressed as [42, 43]:

$${\text{f}}_{\text{t}}=\sigma \left({\text{W}}_{\text{f}}\cdot \left[{\text{h}}_{\text{t}-1},{\text{x}}_{\text{t}}\right]+{\text{b}}_{\text{f}}\right)$$ (10)

$${\text{i}}_{\text{t}}=\sigma \left({\text{W}}_{\text{i}}\cdot \left[{\text{h}}_{\text{t}-1},{\text{x}}_{\text{t}}\right]+{\text{b}}_{\text{i}}\right)$$ (11)

$${\tilde{\text{C}}}_{\text{t}}=\text{tanh}\left({\text{W}}_{\text{C}}\cdot \left[{\text{h}}_{\text{t}-1},{\text{x}}_{\text{t}}\right]+{\text{b}}_{\text{C}}\right)$$ (12)

$${\text{C}}_{\text{t}}={\text{f}}_{\text{t}}\cdot {\text{C}}_{\text{t}-1}+{\text{i}}_{\text{t}}\cdot {\tilde{\text{C}}}_{\text{t}}$$ (13)

$${\text{h}}_{\text{t}}={\text{o}}_{\text{t}}\cdot \text{tanh}\left({\text{C}}_{\text{t}}\right)$$ (14)

Here, f_t_, i_t_, and o_t_ represent the Forget Gate, Input Gate and Output Gate, respectively. Besides, σ represents sigmoid function, C_t_ represents the cell state update value at time t, ${\tilde{\text{C}}}_{\text{t}}$ represents the candidate state value of the input cell at time t. W_f_, W_i_, W_C_ represent the weight of Forget Gate, Input Gate and Output Gate.

### Performance measures

The fitting and prediction accuracy can be employed by the mean absolute error (MAE), root mean square error (RMSE) and mean absolute percentage error (MAPE) [22]. The smaller the error is, the better the fitting effect [19]. In this paper, MAE, RMSE, and MAPE are applicable to evaluating the fitting and prediction accuracy of the three models, respectively, which are expressed as:

$$\text{MAF}=\frac{\sum_{\text{t}=1}^{\text{n}}\left|{\text{X}}_{\text{t}}-{\hat{\text{X}}}_{\text{t}}\right|}{\text{n}}$$ (15)

$$\text{RMSE}=\sqrt{\frac{\sum_{\text{t}=1}^{\text{n}}{\left({\text{X}}_{\text{t}}-{\hat{\text{X}}}_{\text{t}}\right)}^{2}}{\text{n}}}$$ (16)

$$\text{MAPE}=\frac{\sum_{\text{t}=1}^{\text{n}}\left|\frac{{\text{X}}_{\text{t}}-{\hat{\text{X}}}_{\text{t}}}{{\text{X}}_{\text{t}}}\right|\times 100}{\text{n}}$$ (17)

Where the ${\hat{\text{X}}}_{\text{t}}$ is the predicted value, X_t_ is the actual value and n is the sequence sample size [19].

### Data processing and analysis

The data of this study was recorded with EXCEL 2010, and the ARIMA was performed using SPSS23.0 software, and Matlab 2020b was adopted to construct the GM(1,1) and LSTM models. Significant level is 0.05.

## Results

### Trends of TB cases in mainland China

**Fig 2:**
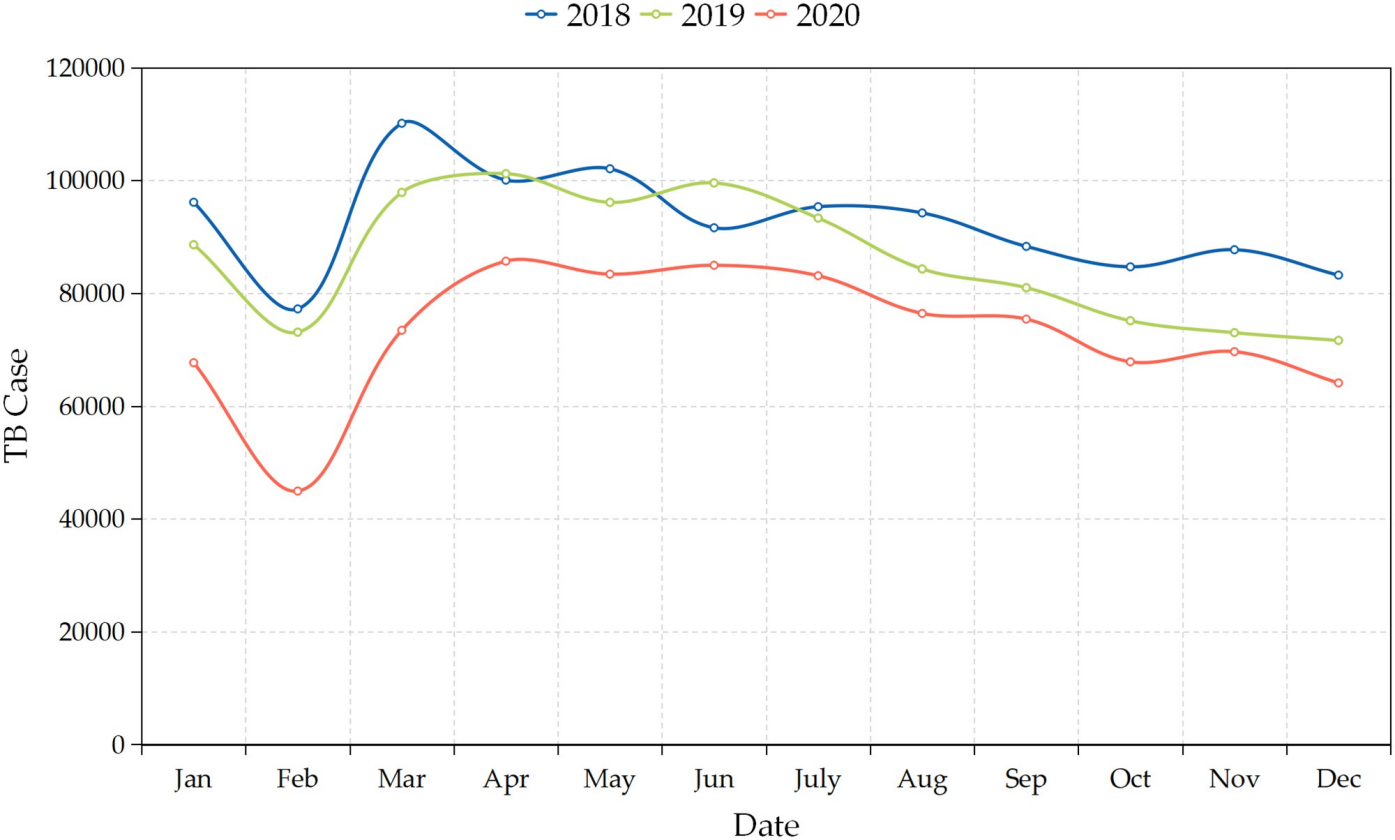
Time series monthly reported cases of TB in mainland China from January 2018 to December 2020.

There were 3,021,995 TB cases in mainland China from January 2018 to December 2020. As is shown in Fig 2, the overall TB cases data in mainland China take on a downtrend trend. In a year, the monthly TB cases data presents lowest level in January and February, whereas March and April, in contrast, has the highest level. Simultaneously, it may be observed that the monthly TB cases data has roughly seasonal fluctuations.

### ARIMA model

**Fig 3:**
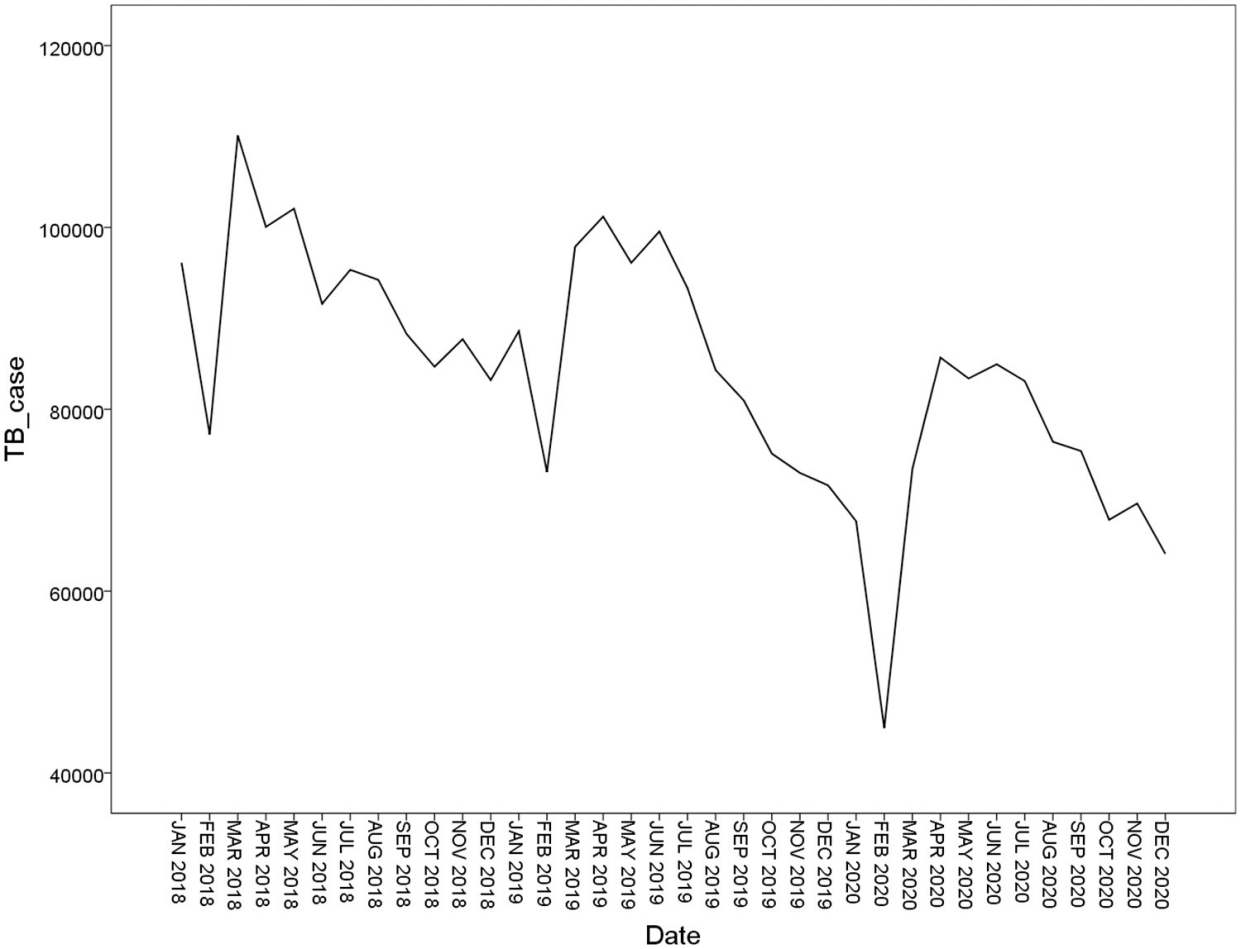
Plot of time series of original sequence.

**Fig 4:**
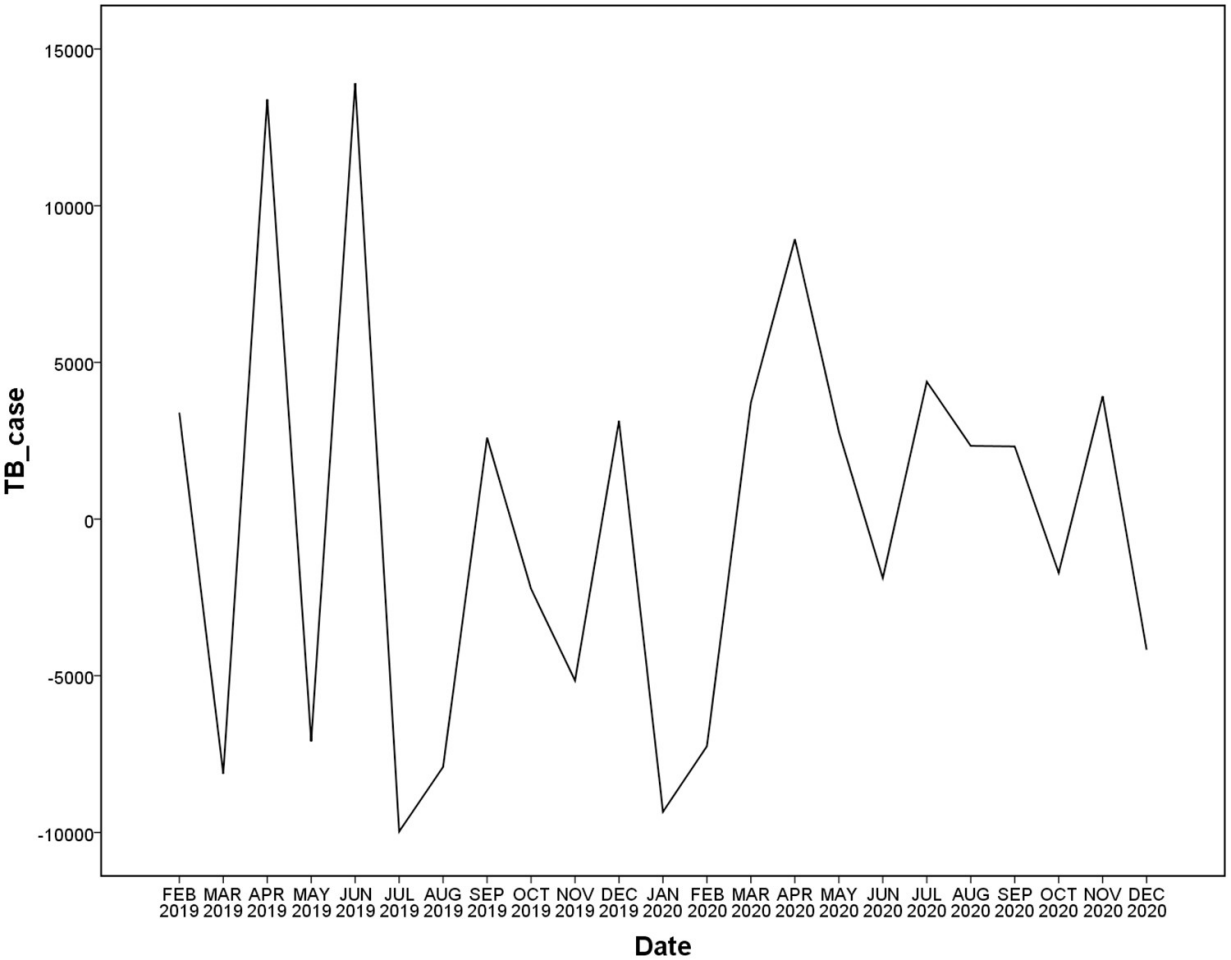
Plot of time series of after a first-order difference and a first-order seasonal difference.

In our study, the SARIMA was selected due to that the data of TB cases has roughly seasonal fluctuations. The basic optical requirement of the ARIMA model is stationary data. As is shown in Fig 3, the original sequence shows non-stationary time series, so the trend difference and the seasonal difference were processed in order to eliminate data instabilities. After a first-order difference and a first-order seasonal difference (Fig 4), the time series were stationary, thus the parameters *d* and *D* were 1, respectively. To confirm the estimation of ARIMA model parameters of *q*, *p*, *Q*, and *P*, the auto-correlation function (ACF) and partial auto-correlation function (PACF) graphs were performed.

**Fig 5:**
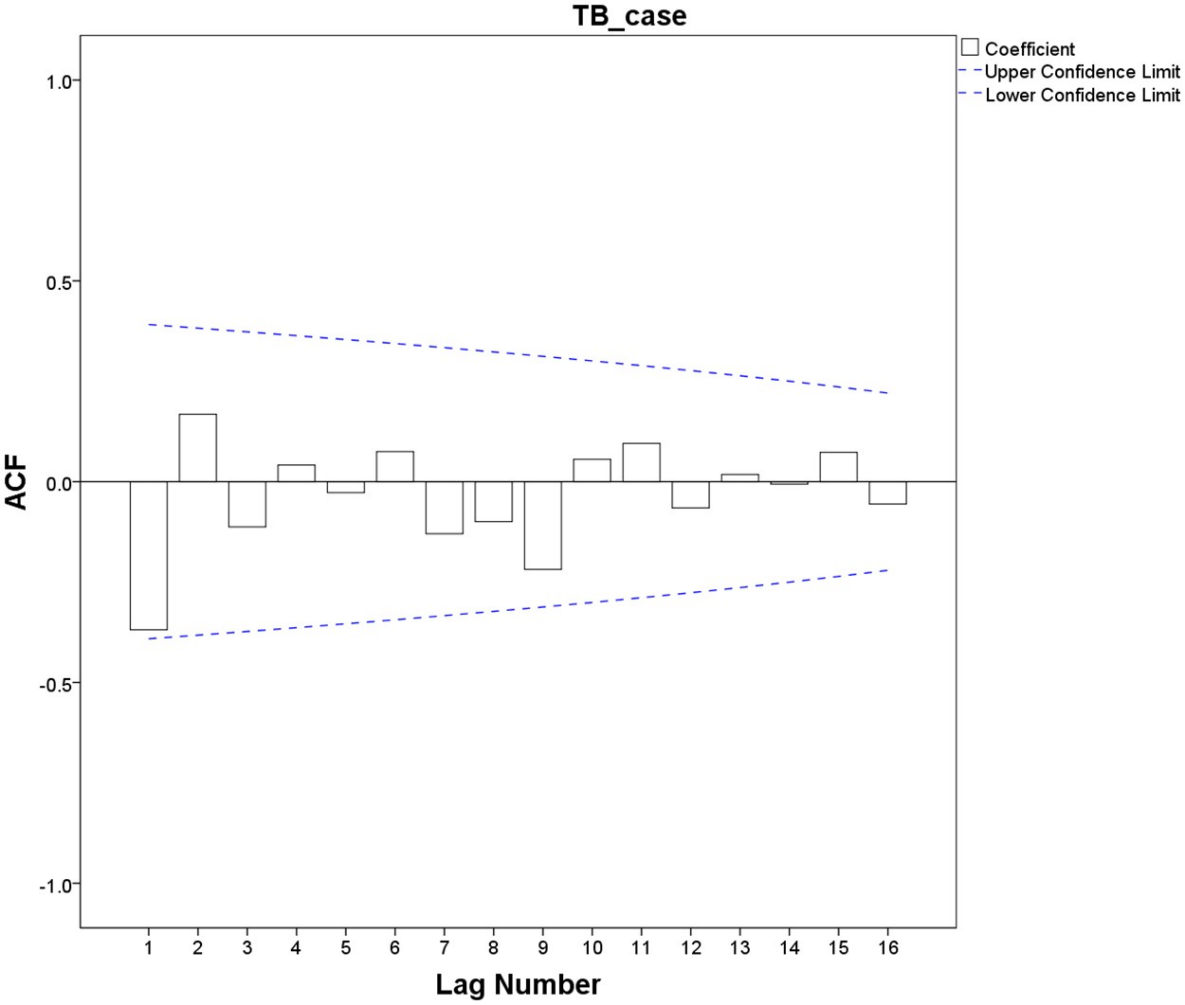
Plot of ACF after a differenced TB cases time series.

**Fig 6:**
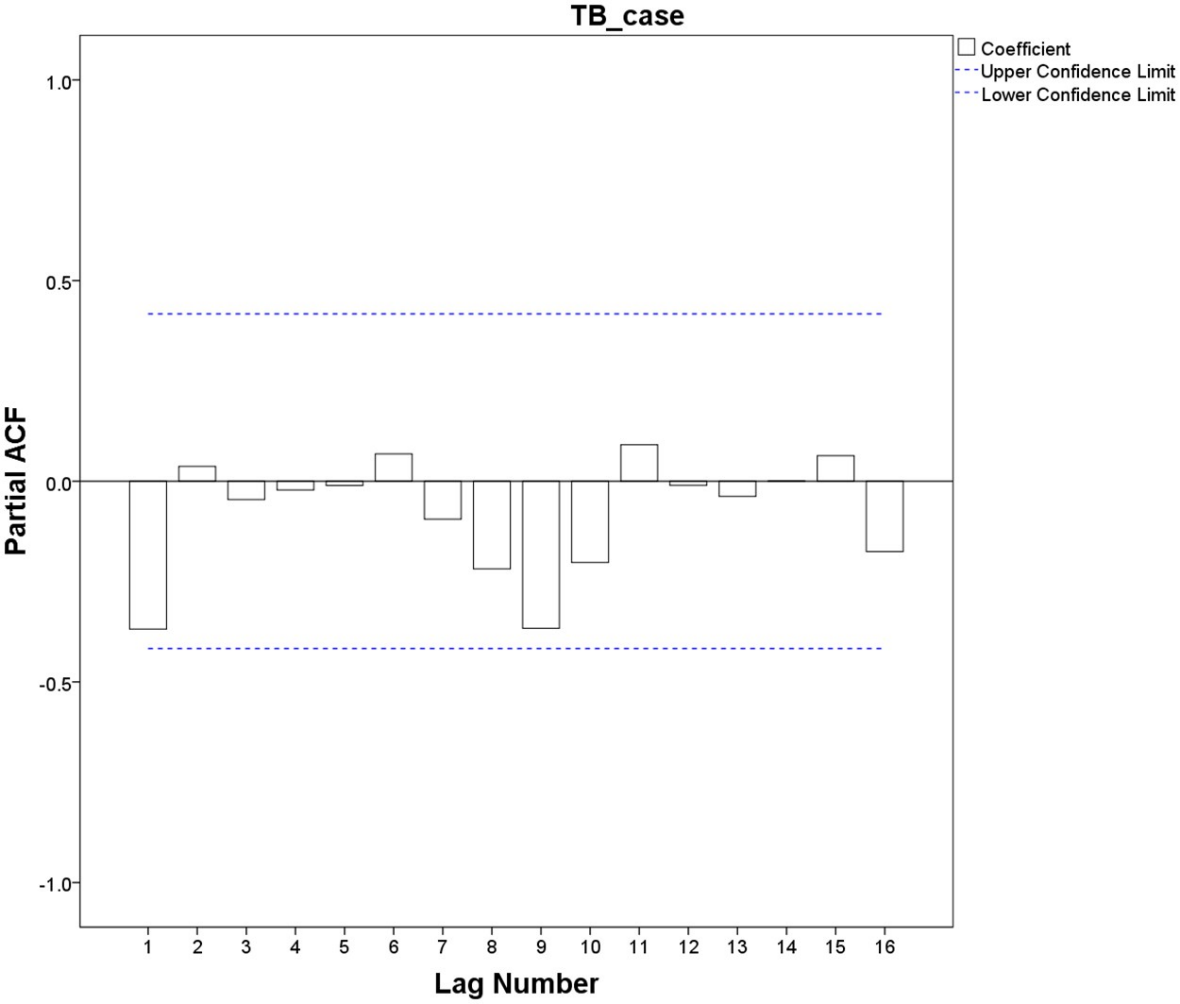
Plot of PACF after a differenced TB cases time series.

Both ACF and PACF graphs showed that the sequence presented high seasonal characteristics with a circle of 12, indicating the parameter of *s* was 12 (s = 12). The ACF graph showed that after a first-order difference (Fig 5), the values of auto-correlation coefficients did not exceed ± 2 times the estimated standard deviation range, and the peak was at lag 0, which was initially identified as the parameters of *q* was 0, p was 0 or 1. Similarly, the PACF graph was represented after a first-order seasonal difference (Fig 6), the partial correlation coefficient did not exceed the range of ± 2 times the estimated standard deviation, and it is initially identified that *P* was 0, Q was 0, or 1.

Further, the candidate ARIMA models were based on both the preliminary parameters values and the modeling estimation and diagnosis, which were selected as ARIMA (0,1,0) × (0,1,1)_12_, ARIMA (0,1,0) × (0,1,0)_12_, ARIMA (1,1,0) × (0,1,0)12 and ARIMA (1,1,0) × (0,1,1)_12_. The results are shown in Table 3.

**Table 3:** Parameter estimation of candidate ARIMA models.

ARIMA Modes	R^2^	BIC	Ljung-Box (Q) test	
			Q-Statistics	*p*-value
ARIMA (0,1,0) × (0,1,1)_12_	0.73	17.97	12.48	0.77
ARIMA (0,1,0) × (0,1,0)_12_	0.73	17.79	12.62	0.81
ARIMA (1,1,0) × (0,1,0)_12_	0.77	17.82	9.60	0.92
ARIMA (1,1,0) × (0,1,1)_12_	0.77	18.00	9.39	0.89

By the diagnostic checking of residuals with the Ljung-Box (Q) test, we find that the Q-statistics of four models were all bigger than 0.8 and the *p*-values were all bigger than 0.05 (Table 3), indicating that these models are all adequate and the white noise sequence. To obtain the optimal model, the lowest BIC values were observed in four models. As a result, the ARIMA (0,1,0) × (0,1,0)_12_ was the optimal model, whose BIC value was the lowest (17.79) and its residual was white noise sequence, the Q-statistics was 12.62 and *p*-value was 0.81 (Table 3).

### GM(1,1) model

The results show that the parameters of model a was 0.01 and u were 99740, the equation for the GM(1,1) model as follows: X(k+1) = -10057053.55e^(-0.01k)^+10153178.55, the Mean square deviation ratio C value was 0.49, and the Small probability of error P was 0.94.

According to the results in (Table 1), it can ensure that the GM(1,1) model we established is Level 2 (Qualified), and it can be adopted in extrapolated prediction. Besides, the GM(1,1) model parameter of *a* was 0.01, and -*a*≤0.3, which demonstrated the Medium- and long-term prediction can be predicted.

### LSTM model

In this section, we build an LSTM model, which consists of three parts, that is, an input layer, a hidden layer, and an output layer. In the LSTM modeling process, the TB cases data from January 2018 to December 2020 were divide into two parts, 2/3 percent of which is the training set and the rest (1/3) is the test set. We set epochs, learning rating with 60, 0.01, respectively. The loss function uses mean_squared_error, and the optimizer uses Adam. The Look_back is set 12 to find the optimal situation of the current network structure. The results are shown in Table 4.

**Table 4:** The actual values and the prediction results of the three models.

Date	actual values	ARIMA	GM	LSTM
19-Feb	73096	69696	87380	73620
19-Mar	97866	105996	86526	97580
19-Apr	101191	87796	85680	100244
19-May	96106	103200	84842	95929
19-Jun	99555	85646	84013	96138
19-Jul	93318	103290	83192	91464
19-Aug	84304	92212	82379	85603
19-Sep	80973	78374	81573	81252
19-Oct	75123	77351	80776	75207
19-Nov	73000	78152	79986	72188
19-Dec	71631	68496	79204	71270
20-Jan	67682	77023	78430	68097
20-Feb	44933	52181	77663	45219
20-Mar	73427	69703	76904	74241
20-Apr	85684	76752	76152	86788
20-May	83385	80599	75408	83530
20-Jun	84952	86834	74671	86706
20-Jul	83101	78715	73941	82831
20-Aug	76423	74087	73218	72042
20-Sep	75409	73092	72502	66545
20-Oct	67843	69559	71794	60984
20-Nov	69640	65720	71092	53988
20-Dec	64097	68271	70397	53131

### Model comparison

The only 23 values were compared with ARIMA, GM(1,1), and LSTM models, on account of its differencing and seasonal differencing of the ARIMA model, which caused the first 13 values to be lost in the validation set.

In this section, the various statistical tools, such as MAE, RMSE, and MAPE, which used to evaluate the fitting and predicting accuracy. Both the actual values and the predicted values were put them into the formula (15), (16), (17), and then results of MAE, RMSE and MAPE were by calculation as shown in Table 5.

**Table 5:** The values of MAE and MAPE and RMSE of the three models.

Models	MAE	MAPE	RMSE
ARIMA (0,1,0) × (0,1,0)_12_	5638.43	0.0706	22599.46
GM (1,1)	8805.39	0.1210	37452.98
LSTM	2676.08	0.0368	16344.92

**Fig 7:**
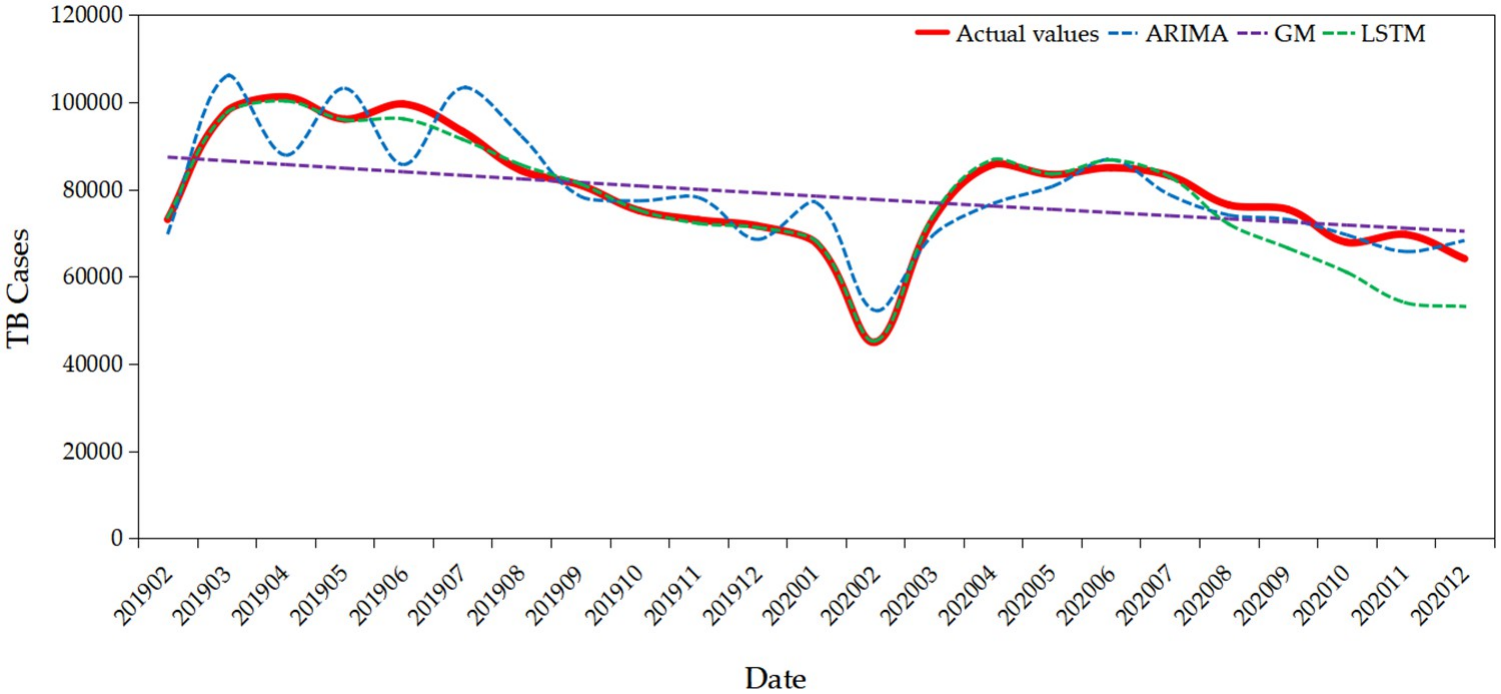
Plot of comparison of predicted values and actual values of three models.

We could see that the MAE, RMSE, and MAPE of LSTM model values were smaller than that of GM(1,1) and ARIMA (0,1,0) × (0,1,0)_12_ model. Additionally, the Fig 7 showed that the predicted value obtained by LSTM can better fit the actual value change trend. Therefore, the LSTM model was the most optimal model, which was more suitable to predict the future trend of TB cases of the mainland in China.

### Prediction

The LSTM model was applied to predict the trend of TB cases in mainland China from January to December in 2021. Predictions results is shown in Table 6.

**Table 6:** Prediction of TB cases in mainland China from January to December in 2021.

Date	Predicted values	Date	Predicted values
21-Jan	60148	21-Jul	75565
21-Feb	37398	21-Aug	68887
21-Mar	65892	21-Sep	67873
21-Apr	78149	21-Oct	60306
21-May	75850	21-Nov	62103
21-Jun	77416	21-Dec	56560

## Discussion

Although TB can be treated effectively, the morbidity and mortality of TB have always been maintained a relative growth trend [4, 44]. It is still a major global public health problem, which poses a serious danger to human health and the development of the society and economy in the world. However, based on a large-scale population and with the rapid development of society and economy in China, the prevention and control of TB is still an important public issue and with difficult challenges. So it still regarded as an important topic in the field of public health in mainland China.

Scientific prediction and analysis of the morbidity and mortality of TB can provide suggestions in public health planning and set the proper policy to adopt effective interventions for this infectious disease [45]. In the field of Disease Surveillance, prediction of the morbidity and mortality infection disease is one of the most important works with higher priorities in public health in China [32]. Upon the above research background, the purpose of this study is to explore the optimized prediction model to predict the epidemic trend of TB cases and propose its prevention and control in mainland China.

Studies have shown that each type of infectious disease prediction model has distinct advantages and disadvantages [46]. It is therefore critical that according to the data characteristics and the sample requirements to create the most suitable prediction model for research objectives. ARIMA is applicable to time series with characteristics of seasonality and periodicity [47]. The GM(1,1) has no special requirements for research data and is also applied in small sample sizes with uncertain time series predictions [17]. While the LSTM model is more suitable for time series with missing values and where there may be a lag of unknown duration [42]. Yet, according to characteristics of the data the sample requirements to construct prediction models is also a precondition for obtaining accurate research results. In our study, TB cases data with 36 samples from January 2018 to December 2020 in mainland China presents characteristics of the seasonality and periodicity, which are fully meet the requirements of ARIMA, GM(1,1), and LSTM models. Therefore, in terms of prediction technique we selected is correct and reasonable.

Further analysis, with the advantage of its structured modeling basis and acceptable forecasting performance, the ARIMA model is the most commonly used technique in time series prediction analysis [4]. Besides, the ARIMA model also can be taken various influencing factors of infection as well as the complicated interactions into consideration in the modeling process [47]. In this study, the seasonal auto regressive integrated moving average (SARIMA) model was applied to predict TB cases, due to the fact that the research data showed evidence of seasonal tendency.

GM(1,1) model was proposed by Deng J. L in 1982, which is widely used in the field of population [48], economic [49], environment [50], power industry [51], medicine [11], etc. This theory is that a system can be defined with a color that represents the amount of clear information about that system [11]. If the information is entirely unknown, it is called a black system. If on the contrary, it is called a white system. Moreover, if some information is known, unknown and/or incomplete, it is called a grey system. In reality, every system can be viewed as a grey system because that its information is known, unknown and/or incomplete. Provided that there are four sets of data, the model can be fitted. So in this paper, the prediction of TB cases can be seen as a grey system, and the monthly TB cases data from January 2018 to December 2020 with 36 samples can be regarded as known information that seeks its changing laws to predict the future state of TB cases.

LSTM model has been widely used to predict infectious disease incidence, such as HIV [15], hepatitis E [52], Dengue fever [53], hand, foot and mouth disease (HFMD) [54], COVID-19 [39], and so on. It is focused on resolving the issues of vanishing gradient [52]. LSTM model has a strong nonlinear mapping capability, which is applicable to process complex issues with long-term dependencies [55]. It can train satisfactory network models by learning from a given sample of data and is suitable for cases where sequence laws are very long time lags of unknown size [15]. Therefore, the LSTM model has a strong capability to address various prediction issues, especially infectious disease prediction.

Model comparison results showed that the MAE, RMSE, and MAPE of LSTM model values were smaller than that of GM(1,1) and ARIMA models. We can deduce that the fitting and predicting performance of the LSTM model was better than that of the ARIMA and GM(1,1) models. The reasons are as follows: first, GM(1,1) and ARIMA models have their disadvantages, for example, they can not extract nonlinear relationships in time series. Second, unlike the other machine-learning based forecasting models, the LSTM model overcomes the shortcomings in vanishing gradient during the training process. Third, the LSTM model is more tolerant to the data and is less vulnerable to model misspecification issues than other time series prediction models. Meanwhile, compared with the GM(1,1) and ARIMA models, it is a more effective deep learning model for continuous data. Thus, the LSTM model has good performance in prediction for infectious disease.

As a result, the LSTM model was applied to predict the TB cases in mainland China from January to December in 2021. The study showed that the predicted values of TB cases present fluctuating and the overall trend is decreasing. However, in practice, the morbidity of TB is the comprehensive effect of manifold causes. The COVID-19 pandemic could affect TB control programs worldwide due to the impairing TB diagnosis and treatment [56, 57], China is no exception. Moreover, although the prevention and control of the COVID-19 pandemic is more effective in China, it also can present great challenges for the medical service system and medical care capacity. Therefore, it must be noted that we still can not ignore the occurrence of an outbreak of TB cases in mainland China in the future.

To the best of our knowledge, this is the first study to construct the ARIMA, GM(1,1), and LSTM models for the prediction of TB cases in mainland China at present. The results showed that the accuracy performance of the LSTM model was higher than that of ARIMA and GM (1,1) models, and the LSTM model can give a credible predictions for TB control and prevention.

However, there are several limitations in this study. One of the limitations is that although the LSTM model has a strong nonlinear mapping capability, the social, cultural, economic, and other factors can not be taken into account in the modeling process [47]. Another limitation is that the prediction results of the LSTM model can act as predictive tools for TB control and prevention, but it should not be used as an exclusive policy-making reference frame due to unexpected emergencies, for example, the COVID-19 pandemic. Since COVID-19 occurred at the end of 2019 in China, missing reports on TB cases are possible to occur in 2020. It results in some inaccuracies in predictions of TB cases to some extent. Therefore, in further work, we will take the influencing factors of TB incidence into the prediction model and continually update data of TB cases so that we can achieve a more suitable and accurate model for its control and prediction.

## Conclusion

In this study, we collected the TB cases from January 2018 to December 2020 in mainland China. Based on the data characteristics and the sample requirements, the ARIMA, GM(1,1), and LSTM models were constructed and compared. The fitting and predicting performance of the LSTM model was better than that of the ARIMA and GM (1,1) models. The results of this study can provide policy advice by the government in mainland China.

## Supporting information

S1 File The monthly TB cases data from January 2018 to December 2020. (DOC)

S1 Data (XLS)
