# Supplementary material for: The research of ARIMA, GM(1,1), and LSTM models for prediction of TB cases in China
Source: PLoS One. 2022 Feb 23;17(2):e0262734. doi: 10.1371/journal.pone.0262734 (PMC8865644; doi:10.1371/journal.pone.0262734)
Supplement: S1 File — (DOC) [file pone.0262734.s001.doc]

| **No** | **Date** | **TB cases** |
| --- | --- | --- |
| 1 | 201801 | 96125 |
| 2 | 201802 | 77224 |
| 3 | 201803 | 110124 |
| 4 | 201804 | 100054 |
| 5 | 201805 | 102063 |
| 6 | 201806 | 91603 |
| 7 | 201807 | 95338 |
| 8 | 201808 | 94232 |
| 9 | 201809 | 88302 |
| 10 | 201810 | 84680 |
| 11 | 201811 | 87709 |
| 12 | 201812 | 83205 |
| 13 | 201901 | 88597 |
| 14 | 201902 | 73096 |
| 15 | 201903 | 97866 |
| 16 | 201904 | 101191 |
| 17 | 201905 | 96106 |
| 18 | 201906 | 99555 |
| 19 | 201907 | 93318 |
| 20 | 201908 | 84304 |
| 21 | 201909 | 80973 |
| 22 | 201910 | 75123 |
| 23 | 201911 | 73000 |
| 24 | 201912 | 71631 |
| 25 | 202001 | 67682 |
| 26 | 202002 | 44933 |
| 27 | 202003 | 73427 |
| 28 | 202004 | 85684 |
| 29 | 202005 | 83385 |
| 30 | 202006 | 84952 |
| 31 | 202007 | 83101 |
| 32 | 202008 | 76423 |
| 33 | 202009 | 75409 |
| 34 | 202010 | 67843 |
| 35 | 202011 | 69640 |
| 36 | 202012 | 64097 |
